# Rectal Involvement of Recurrent Buschke-Lowenstein Tumor Causing Subileus: a Case Report

**DOI:** 10.4021/gr316w

**Published:** 2011-07-20

**Authors:** Savas Yakan, Fevzi Cengiz, Kemal Emre Telciler, Murat UZ, Ali Galip Denecli

**Affiliations:** a Department of Surgery, Izmir Bozyaka Education and Research Hospital, Izmir, Turkey

**Keywords:** Buschke-Lowenstein tumor, Rectal involvement, Recurrence, Subileus

## Abstract

We present a 48-year-old caucasian woman presenting with anal pain, discharge and difficulty in defecation due to recurrent Buschke-Lowenstein tumor with rectal involvement discuss it in the light of literature. A 48-year old caucasian woman was referred to our institute with anal mass causing pain, discharge and difficulty in defecation. She initially had simple excision and electrocoterisation 3 and 15 years before at different centers. At physical examination, multiple vegetative mass lesions presented as a cauliflower-like tumor were seen at perianal region. Colonoscopy showed an inflamated, vegetative mass covering all mucosa annularly and starting from 2 cm away from anal verge and reaching until 20 cm was seen. Due to the large extent of tumor invasion in this case, curative surgery would have been achieved only by wide local surgical excision and abdominoperineal resection due to rectal involvement. This severe mutilation was refused by the patient. Thus, patient was referred to medical oncology for radiochemotherapy. Wide radical excision of Buschke-Lowenstein tumor (BLT) is the preferred treatment for achieving local control, but excision alone often is ineffective treatment. Abdominoperineal resection is necessary in cases with infiltration involving the sphincter muscles or rectum, especialy for recurrent cases.

## Introduction

A Buschke-Lowenstein tumor (BLT; also known as a giant condyloma acuminatum, carcinoma-like condyloma, giant malignant condyloma) was first described by Buschke and Loewenstein as penile condyloma like carcinoma without true microscopic invasion findings [1]. It may develop both in the penis and anorectal region and female genital organs [2]. It is a rare sexually transmitted disease; the incidence is probably 0.1% in the general population. Although its histological appearance is usually of a benign tumor and it is associated with less-oncogenic strains of a human papilloma virus, there is evidence that progression to malignancy can ocur [3].

In this article, we present a 48-year-old caucasian woman presenting with anal pain, discharge and difficulty in defecation due to recurrent BLT with rectal involvement which was locally excised 3 and 15 years ago in different centers and discuss it in the light of current literature.

## Case Report

**Figure 1:**
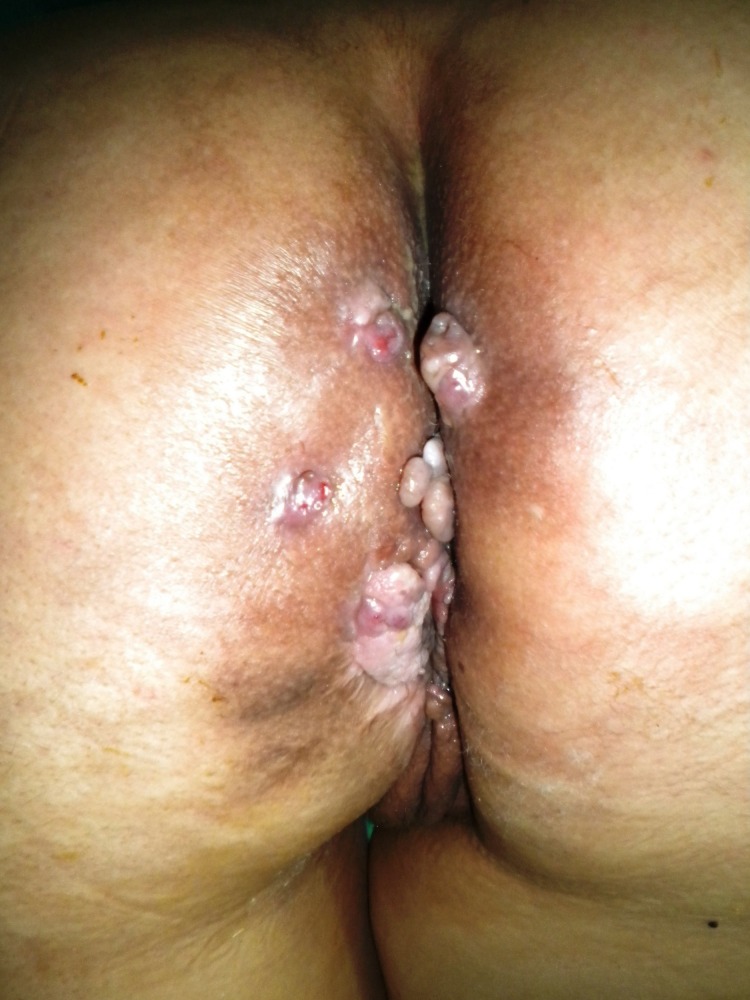
Multiple vegetative mass lesions presented as a cauliflower-like tumor were seen at perianal region causing multiple perianal fistula formation.

**Figure 2:**
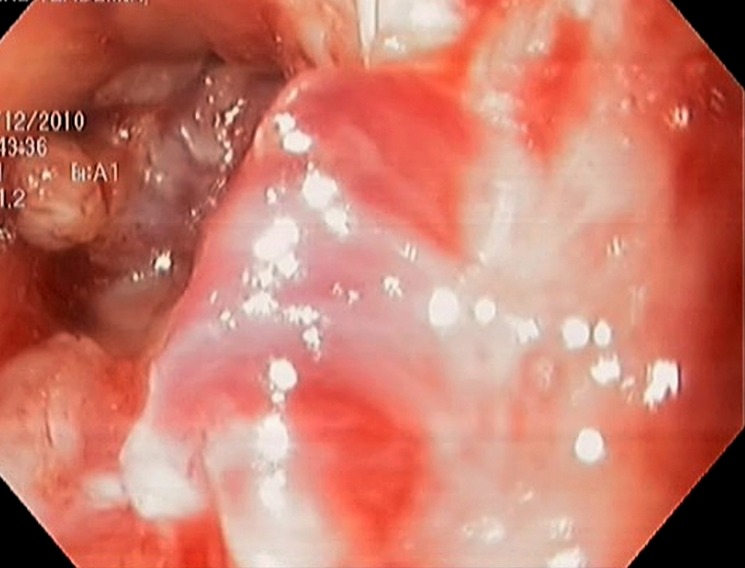
Colonoscopic appearance an inflamated, vegetative mass covering all mucosa annularly and starting from 2 cm away from anal verge and reaching until 20 cm.

A 48-year old caucasian woman was referred to our institute with anal mass causing pain, discharge and difficulty in defecation. This lesion had been increasing slowly for more than sixteen years. She initially had simple excision and electrocoterisation 3 and 15 years before at different centers. At physical examination, multiple vegetative mass lesions presented as a cauliflower-like tumor were seen at perianal region, the biggest one sizing 5 x 3 cm, causing multiple perianal fistula formation (Fig. 1), it was painful with touching during digital rectal examination. Obliteration was present at anal canal. No enlarged inguinal lymph node was palpated bilaterally. The patient had no detectable immunological defect and had no symptoms of acquired immunodeficiency syndrome. Chest x-ray revealed no suspicious lesion. Routine admission laboratory results were within normal limits. Colonoscopy showed obliteration in anal canal due to mass lesions. Rectum, rectosigmoid region and sigmoid colon was seen and colonoscope was pushed forward until descending colon. An inflamated, vegetative mass covering all mucosa annularly and starting from 2 cm away from anal verge and reaching until 20 cm was seen (Fig. 2). It was not allowing the colonoscope to pass through and could only be passed with thin endoscope. Multiple biopsies were taken. The pathologic analysis of the specimens revealed multiple foci of squamous cells of epithelium with severe dysplasia and coilocytosis. Taken together, these findings were consistent with the diagnosis of recurrent BLT of rectal involvement. Due to the large extent of tumor invasion in this case, curative surgery would have been achieved only by wide local surgical excision and abdominoperineal resection due to rectal involvement. This severe mutilation was refused by the patient. Thus, patient was referred to medical oncology for radiochemotherapy.

## Discussion

BLT is a rare sexually transmitted disease, triggered by human papilloma virus (HPV), usually genotype 6 or 11 [1]. Risk factors for HPV transmission are: multiple partners, homosexuality, lack of genital hygiene, and chronic genital infections. It is regarded as a cauliflower-like exophytic giant tumor in the genital or anorectal regions and its pathogenesis and natural history have not been well described. Most common presenting symptoms are pelvic pain, perianal discharge, anorectal bleeding or disturbance of defecation [4]. They have propensity for fistula formation, infection and malignant transformation.

BLT has been reported to transform into malignant cancer in 30-50% of patients. It remains unknown which viral factors (e.g., an increase of viral expression) or host factors (e.g., a decreased cellular immune response) may affect the oncogenic potential of HPV to cause the giant condyloma to turn squamous cell carcinoma (SCC). The mean time of malignant transformation from benign BLT to histologic malignancy is approximately five years [4]. Differentiation between BLT and malignant SCC mainly depends on the lack of histologic invasion, infrequent mitotic figures, and no evidence of metastases. Despite a benign histology and low risk of metastasis, it is considered as an intermediate lesion between condyloma acuminata and SCC [5].

There are still many problems relevant with the treatment for BLT because of the high recurrence rates (up to 50%) and the lack of adequate series of patients following the same procedure [4]. Surgical excision has been the most common form of treatment regardless of the type of surgery (local or radical excision). Abdominoperineal resection, which should be performed in cases of tumor recurrence, infiltration involving the sphincter muscles or rectum and malignant transformation.

Radiation therapy and topical or systemic chemotherapy without surgery have also been used with minimal response, and should only be considered as palliative treatment in patients that present with nonresectable tumours [4, 6]. Sometimes it is recommended that reduction of the tumoral mass through radiotherapy or chemotherapy should precede surgical excision.

The most important problems with the treatment of BLT are recurrence after inadequate surgery, anorectal involvement and malignant transformation; as it is mentioned in the literature and as in our case. In our patient, the initial simple excision and electrocoterisation by the first surgeon was inadequate, which caused recurrent BLT twelve years later. Another surgeon (gynecologist) performed local excision three years ago.

As a result, wide radical excision of BLT is the preferred treatment for achieving local control, but excision alone often is ineffective treatment for BLT. Abdominoperineal resection is necessary in cases with infiltration involving the sphincter muscles or rectum, especialy for recurrent cases.
